# Stirred Yogurt as a Delivery Matrix for Freeze-Dried Microcapsules of Synbiotic EVOO Nanoemulsion and Nanocomposite

**DOI:** 10.3389/fmicb.2022.893053

**Published:** 2022-05-19

**Authors:** Hoda S. El-Sayed, Khamis Youssef, Ayat F. Hashim

**Affiliations:** ^1^Dairy Science Department, Food Industries and Nutrition Research Institute, National Research Centre, Giza, Egypt; ^2^Agricultural Research Center, Plant Pathology Research Institute, Giza, Egypt; ^3^Agricultural and Food Research Council, Academy of Scientific Research and Technology, Cairo, Egypt; ^4^Fats and Oils Department, Food Industries and Nutrition Research Institute, National Research Centre, Giza, Egypt

**Keywords:** microencapsulation, extra virgin olive oil, nanoemulsion, nanocomposite, probiotic strains, stirred yogurt

## Abstract

Nowadays, dairy products are considered a good matrix to deliver many functional substances either vital oils or probiotic cells. Two models of microcapsules were produced from co-encapsulation of extra virgin olive oil (EVOO) nanoemulsion or nanocomposite and synbiotic bacteria (maltodextrin with *Lactobacillus acidophilus* and *Bifidobacterium bifidum*) using the freeze-drying technique. These models of microcapsules were added to stirred yogurt, and then its storage effect on microbiology, chemically, and sensory properties were evaluated for 21 days. The average droplet size and zeta potential distribution of EVOO nanoemulsion and nanocomposite were investigated. Also, oxidative stability, microencapsulation efficiency, release profile, and antioxidant activity were studied. The results showed that the average particle size of EVOO nanoemulsion and nanocomposite ranged between 416 and 475 nm, while zeta potential was −39.6 and −33.6 mV, respectively. The induction period of EVOO extracted from nanoemulsion and nanocomposite microcapsules models was 11.30 and 8 h. The microencapsulation efficiency of probiotic and EVOO was determined at 88.84 and 65.61% for the nanoemulsion microcapsules model, while the nanocomposite microcapsules model showed 98.49 and 72%. The two models of microcapsules have boosted the viability of probiotic bacteria inside stirred yogurt than free cells. Also, the presence of microcapsules did not affect the viability of stirred yogurt starter cultures, and high values for the total solid and protein were detected. Therefore, the results recommended that stirred yogurt is a good delivery carrier for highly antioxidant and healthy microcapsules of synbiotic EVOO nanoemulsion and nanocomposite.

## Introduction

Extra virgin olive oil (EVOO) has rapid digestion, nutritional, therapeutic, and economic properties. EVOO is a source of phenolic compounds with a powerful antioxidant activity that includes hydroxytyrosol, tyrosol, and secoiridoid derivatives (Francis et al., 2019). Despite all the proven potentiality, it has poor storage stability, especially upon exposure to oxygen, light, moisture, and high temperature (Calvo et al., 2012). Oxidative degradation results in a loss of nutritional quality and the development of undesired flavors. Therefore, it affects the shelf stability and sensory properties of the oil (Bendini et al., 2006; Koç et al., 2015).

As well, consumer demand for functional foods with probiotics is increasing because of the health benefits (Min et al., 2019). Generally, probiotics are live microorganisms, when ordered in satisfactory amounts, give the host health benefit (Morelli and Capurso, 2012). Regular consumption of 10^7^-10^8^ log CFU/ml of probiotics is recommended to confirm its positive healthy functions in the human body (Terpou et al., 2019). Several types of probiotic bacteria may be affected by the condition of processing as high temperatures, pH values, low water activity, and storage time (Shah et al., 1995; Tripathi and Giri, 2014). Besides, probiotic bacteria are also exposed to enzymes, stomach acids, and bile salts during their passing through the gastrointestinal tract, which adversely affects the survivability of these bacteria (Kailasapathy and Chin, 2000; Raghuwanshiet et al., 2018).

From the previous points, microencapsulation technology is considered a promising technique to increase self-life during olive oil manufacturing (Calvo et al., 2012), prevent losing healthy properties as well as the viability of probiotic bacteria (Mahmoud et al., 2020). In addition, microencapsulation of olive oil has the opportunity to improve economic value, like healthy food, and may provide a therapeutic purpose. Microencapsulation was able to protect the core materials or sensitive cells and substances from adverse conditions by surrounding these substances or bacterial cells with coating materials (Razavi et al., 2021). So, the encapsulated ingredients can arrive in the specific target area without being undesirably affected by environmental influences.

Several encapsulating materials have been used for the microencapsulation techniques, including polysaccharides (sodium alginate, carrageenan, pectin, chitosan, xanthan, maltodextrin, and dextrin), proteins (whey protein, casein, soy protein, and gelatin). It was observed that sodium alginate was not stable in the acidic conditions, so it has poor protection to the entrapped materials in this condition. This limits its application as a coating material to enhance this disadvantage it was added or mixed with other materials like whey protein, dextrin, or chitosan (Mahmoud et al., 2020). Whey protein as coating material protected the entrapped substances, especially probiotic cells from the acidic conditions in the stomach (Doherty et al., 2011), fish oil (Aghbashlo et al., 2013), and linseed oil (Gallardo et al., 2013). Additionally, prebiotic agents like maltodextrin could be used for two purposes: capsulation material and prebiotic agents to activate entrapped probiotic cells. Also, maltodextrin is normally used as wall material in microencapsulation according to its low viscosity at high-solid concentration, good solubility, good protection, low cost, neutral aroma, and taste (Madene et al., 2006). However, it is usually used with other coating materials (such as whey protein and sodium alginate) for offering efficient and effective microencapsulation because it is poor emulsifying. Also, Bentonite is natural clay composed of montmorillonite, which is a 2:1 type aluminosilicate in the smectite group. Dong et al. (2021) prepared and tuned alginate-bentonite microcapsules by extrusion and emulsification. Hamed et al. (2020) developed nanocomposite microspheres based on alginate/chitosan to deliver vehicles of omega-3-rich oils. Nanocomposite microcapsules have a good surface structure, a higher biodegradability rate, and better protection from active chemicals than polymeric nanocomposites (Arjona et al., 2021).

Dairy products are considered the major food groups that have been supplemented with probiotic bacteria, such as cheese yogurt and ice cream (Castro et al., 2015; El-Sayed et al., 2015; El-Sayed and El-Sayed, 2020). The food matrix was considered the perfect medium to deliver microencapsulated substances either vital oils or probiotic cells to humans, especially dairy products. Stirred yogurt was considered a favorable dairy product to humans because it is a good source of proteins, vitamins, and minerals. Stirred yogurt can be used as a delivery system to transport vital substances and probiotic cells with sufficient amounts to the humans to donate vital therapeutic effects. Moghanjougi et al. (2021) produced microcapsules of probiotics (*L. acidophilus LA-5 and B. animalis BB-12*) using a freeze-drying technique based on the emulsion method in the sodium alginate and pectin. Calvo et al. (2012) used different wall materials to microencapsulate EVOO by freeze frying system.

Although several studies on microencapsulation of EVOO and probiotic bacteria have appeared in the literature, to our knowledge, this is the first study that was designated to produce two models of freeze-dried nano-in-micro microcapsules based on synbiotic EVOO nanoemulsion and nanocomposite. The main objectives of this study were (i) to apply these models in stirred yogurt as a delivery matrix for EVOO and probiotic bacteria (ii) the effect of adding these microcapsules to stirred yogurt was evaluated during 21 days of storage for microbiology, chemically, and sensory properties, and (iii) the antioxidant activity was evaluated for the two models of microcapsules and after the addition to the stirred yogurt.

## Materials and Methods

### Chemicals

Maltodextrin was purchased from Loba Chemie, Mumbai, India. Extra virgin olive oil (EVOO) was gotten from the oil extraction unit, National Research Centre, Egypt. Nanoclay hydrophilic bentonite was purchased from Sigma-Aldrich (St. Louis, MO, USA). Sodium alginate from brown algae (91%) was received from Lobachemia (India). Whey protein concentrate (WPC) 80% was obtained from Agri-mark, USA. 2,2-diphenyl-1-picrylhydrazyl (DPPH, 90%) was purchased from Sigma (St. Louis, MO, USA). All other solvents and reagents were purchased from various suppliers and used without further purification.

### Microbial Strains

Probiotic strains were obtained from the dairy department, National Research Center as *Bifidobacterium bifidum* NRRL B-41410 and *Lactobacillus acidophilus* CH-2.

### Physicochemical Analysis of EVOO

Peroxide value (PV), iodine value (IV), acid value (AV), and saponification value (SV) were determined according to the standard methods of the [American Oil Chemists' Society (AOCS), 2005; AOCS, 2011]. Specific extinction k_232_ and k_270_ extinction coefficients were calculated from the absorbance at 232 and 270 nm, respectively, with a UV-Vis spectrophotometer (T80 UV/VIS Spectrometer, PG Instruments Ltd., UK), using a 1% solution of oil in cyclohexane and a path length of 1 cm.

### Preparation of Probiotic Cell Suspensions

The probiotic strains (*Lb. acidophilus* and *B. bifidum*) were activated individually to obtain high biomasses by MRS broth (De Man-Regosa-Sharp) and incubated for 24 h at 37°C anaerobically (Fayed et al., 2018). The cell pellets were harvested by centrifugation at 6.000 rpm for 15 min at 4°C. The obtained cells pellets were washed with a sterile saline solution [0.9% (w/v) NaCl] and stored at 8°C for further use.

### Microencapsulation of Synbiotic EVOO Nanoemulsion and Nanocomposite

Synbiotic EVOO nanoemulsion or nanocomposite microcapsules were prepared in three stages using: ultrasonication, blending, and freeze drying techniques as the following:

#### First Stage: Preparation of EVOO Nanoemulsion and Nanocomposite

EVOO nanoemulsion and nanocomposite were prepared using emulsification and the ultrasonication process. Tween 20 (2%) was mixed with distilled water as an aqueous phase using a magnetic stirrer (WiseStir MSH-20A, Korea) at 1.000 rpm. Then, this aqueous phase was added dropwise to the oil phase (10%) while magnetically stirring. The mixture was homogenized with a high-speed homogenizer (X520, CAT Ingenieurburo M. Zipperer, GmbH, Germany) for 3 min at 16.000 rpm to prepare a coarse emulsion. After that, the prepared emulsion was ultrasonicated using a bench-scale sonicator (Sonics and Materials Inc. 53 Church Hill Rd., Newtown, CT, USA) at 60% of full power amplitude (60 W) for 10 min and using a probe with a diameter of 22.5 mm. The same procedure was used for the preparation of EVOO nanocomposites. In a brief, a suspension of nano-clay hydrophilic bentonite (5 g) was stirred overnight at 30°C in distilled water (250 ml). Nano-clay suspension (50 ml) was mixed to the EVOO-dispersed phase for 2 h using a mechanical stirrer before the emulsification process. The preparation of W/O nanocomposite was ultrasonicated using a bench-scale sonicator.

#### Second Stage: Preparation of Synbiotic EVOO Nanoemulsion and Nanocomposite

Whey protein concentrate (WPC) solution was dispersed into deionized water overnight at room temperature for complete hydration. Sodium alginate (SA) and WPC in a ratio of 1:1 were mixed under continuous stirring. Synbiotic was prepared by blending previously prepared probiotic cell suspensions (25%) with maltodextrin biopolymer (3%). Both SA-WPC and synbiotic suspensions were blended and homogenized with EVOO nanoemulsion or nanocomposite using a high-speed homogenizer (Ingenieurbüro CAT, Germany) at 16.000 rpm for 1 min.

#### Third Stage: Preparation of Two Models of Microcapsules Based on Nanoemulsion and Nanocomposite

**Figure 1:**
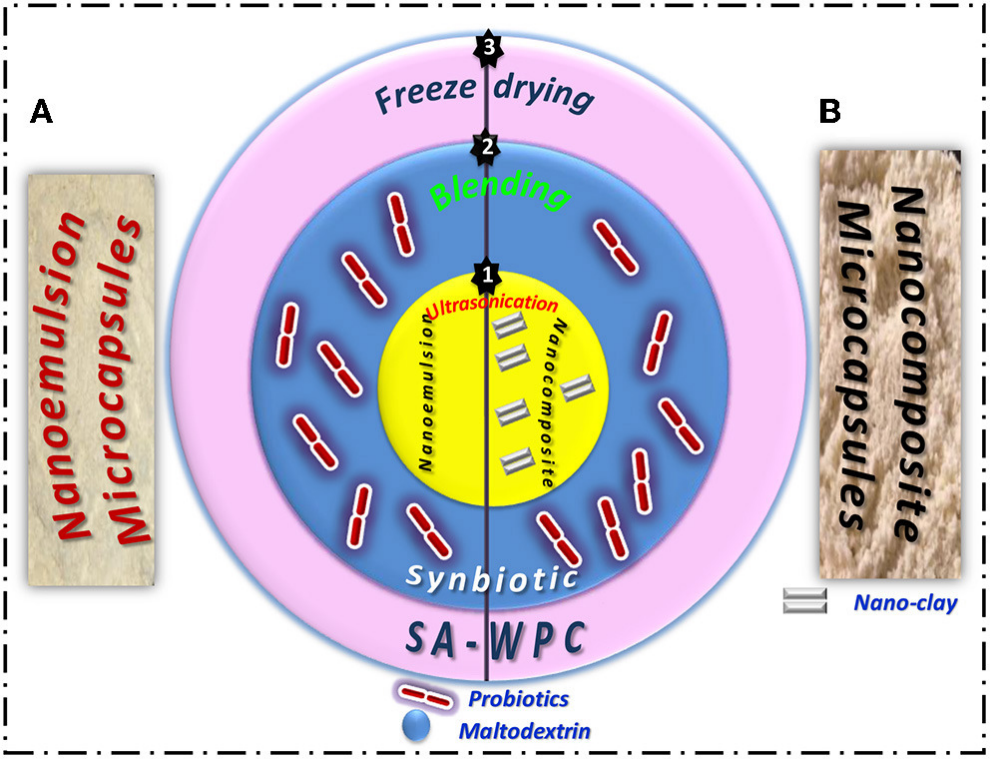
Three stages of freeze-dried synbiotic EVOO **(A)** nanoemulsion and **(B)** nanocomposite microcapsules preparation.

The synbiotic EVOO nanoemulsion and nanocomposite mixture was frozen overnight at −80°C and then freeze-dried using a Christ freeze dryer (ALPHA 1-4 LSC) for 48 h. Freeze-drying has been performed under optimal conditions for receiving the highest survivability of probiotics. Dried samples were then grounded using a planetary ball mill. The resulting freeze-dried powders were packed in 250-ml sterile bottles and stored at 4°C (Figure 1).

### Particle Sizing and Zeta Potential for EVOO Nanoemulsion and Nanocomposite

Measurement of EVOO nanoemulsion and nanocomposite size (Section. First Stage: Preparation of EVOO Nanoemulsion and Nanocomposite), distributions, and zeta potential was performed by a dynamic light scattering method (DLS) using Zetasizer Nano ZS (Malvern Instruments, UK) at room temperature. Before measurement, 30 μL of the EVOO nanoemulsion or nanocomposite was diluted with 3 ml of water at 25°C. Particle-size data were expressed as the mean of the Z-average of three independent batches of the EVOO nanoemulsion or nanocomposite.

### Oxidative Stability

The oxidative stability of EVOO extracted from nanoemulsion and nanocomposite microcapsules was determined as the induction period (IP, h) using a Rancimat apparatus (Model 892 Professional Rancimat, Metrohm SA, Herisau, Switzerland) as the reference analysis for all olive oil samples. The oxidation reactions were increased by keeping 3 g of oil at 110°C under a constant airflow of 20 L/h, and then determining the conductivity variation of water (60 ml) due to the increase in volatile oxidative compounds.

### Microencapsulation Efficiency (ME)

#### Probiotic Strains

The microencapsulation efficiency of resulting microcapsules was evaluated according to Mahmoud et al. (2020). One gram of the different microcapsules was added to 9 ml of sterile tri-sodium citrate solution (2%, w/v) and stirred to dissolve the microcapsules, considering the first dilution (1:10). After that, prepared serial dilution using normal saline (0.9% NaCl) and added the suitable dilution to plates using the pour plate method and the MRS agar (Man-Regosa and Sharp) medium. The plates were incubated at 37°C for 48 h under anaerobic conditions. The microencapsulation efficiency percentage (ME _probiotic_ %) was calculated as the following:


$$\begin{array}{l} {\text{M}\text{E}}_{\text{probiotic}}\;\%=\frac{{\text{Log}}_{10}\text{N}\;}{{\text{Log}}_{10}{\text{N}}_{0}}\;\text{x}\;100 \end{array}$$

where N = the number of the probiotics cells inside the microcapsules; N_o_ = the number of the free probiotics cells was added to different formulations.

#### Extra Virgin Olive Oil

The surface oil content was measured to calculate the ME_EVOO_%. Surface oil was investigated by a method described by Tonon et al. (2011). Briefly, 50-ml n-hexane was added to 3 g of microcapsules followed by shaking for 2 min at room temperature. The suspension was then filtered through Whatman No. 1 filter paper, and the residue was washed three times with 20-ml n-hexane. Then, the solvent was evaporated until constant weight at reduced pressure. The amount of surface oil was determined by the mass difference between the initial clean flask and that containing the extracted oil residue (Jafari et al., 2008). Total oil content is assumed to equal the initial oil since preliminary tests have shown that all the initial oil was retained, which was expected since the investigated oil is non-volatile. The microencapsulation efficiency percentage (ME_EVOO_ %) was calculated using the following equation:


$$\begin{array}{l} {\text{M}\text{E}}_{\text{EVOO}}\;\%=\frac{\text{Total}\;\text{oil}\;\text{content}-\text{Surface}\;\text{oil}\;\text{content}\;}{\text{Total}\;\text{oil}\;\text{content}}\;\text{X}\;100 \end{array}$$

### Morphological Features

#### Transmission Electron Microscope (TEM)

TEM analysis was carried out to study the structure and morphology of the prepared synbiotic EVOO nanoemulsion and nanocomposite by using a JEOL JEM-1230 transmission electron microscope. Twenty microliters of diluted samples were placed on a film-coated 200-mesh copper specimen grid for 10 min, and the fluid excess was eliminated using filter paper. The grid was then stained with one drop of 3% phosphotungstic acid (PTA) and allowed to dry for 3 min. The coated grid was dried and examined under the TEM microscope (Philips, CM12). Then, samples were observed by operating at 120 kV.

#### Scanning Electron Microscope (SEM)

The morphology of the developed microcapsules was determined by scanning electron microscope (SEM) (SEM, quanta Fei 250 Republic Czech).

### Release Measurements

#### Preparation of Gastrointestinal Solutions

*For stimulated gastric solution*, the pH of saline solution (0.9% NaCl, w/v) was adjusted to 2 and sterilized by autoclave at 121°C for 15 min; after that, 0.3% pepsin enzyme (Group 1 pepsinogens) was added. *For stimulated intestinal solution*, the pH of the 100-ml distilled water contained 0.3% bile salts, 0.65 NaCl, 0.083 KCl, 0.022 CaCl_2_, 0.138 NaHCO_3_ (w/v %) and was adjusted to 7. and sterilized by autoclave at 121°C for 15 min; after that, 1% pancreatic enzyme (amylase, lipase, and protease and made from cows) was added (Chávarri et al., 2010).

#### *In vitro* Survivability and Release Evaluation of Probiotic Strains

Ten grams of each microencapsulated particle and free cells were added separately into 100-ml gastric solution for 2 h. After that time, one gram of each microencapsulated particle and free cells was obtained to calculate the viable probiotic counts after exposure to the gastric solution. The microencapsulated particles and free cells were collected by gently cooling centrifugation (1,500 rpm for 10 min at 4°C) and added to the intestinal solution for 6 h. One gram of each microencapsulated particle was collected after 2-, 4-, and 6-h intervals to calculate the viable counts of probiotics after exposure to the intestinal solution. After collecting the microencapsulated particles, 3% trisodium citrate was used as the first diluted as mentioned before (Section Microbiological Activities of Stirred Yogurt Treatments) and, after that, serially diluted in saline solution. The suitable diluted was plated using an MRS agar medium and incubated anaerobically for 48 h at 37°C (Fayed et al., 2018).

The release of probiotics from the microcapsules was determined by the method described by Lotfipour et al. (2012). Ten grams of each microcapsule and free cells were put in a dialysis bag individually and transferred into 100-ml simulated colonic solution (0.1-M KH_2_PO_4_, pH 7.00), mixed gently, and incubated at 37°C. At 0-, 1-, 2-, 3-, 4-, 5-, and 6-h time intervals, 1 ml of sample was taken and viable counts were enumerated with serial dilution by saline. The suitable diluted was plated using an MRS agar medium and incubated for 37°C for 48 h, anaerobically.

#### *In vitro* Release Evaluation of Extra Virgin Olive Oil

The *in vitro* release profile of the EVOO from the developed microcapsules was determined using the dialysis method for gastrointestinal conditions. In brief, the dialysis bag was soaked in distilled water to remove the preservatives and then rinsed with gastrointestinal solutions. Three milligrams of microcapsules were put into the dialysis bag and suspended in 100-ml gastric solution for 2 h. After this time, the dialysis bags containing microcapsules were picked up and suspended in intestinal solution (100 ml) for 4 h at 37°C under gentle agitation. The amount of oil released was determined using a UV–Vis spectrophotometer. At specific intervals, 3 ml of the buffer was collected for analysis, and each experiment was carried out in triplicate. The time-dependent release study was performed for 6 h.

### Stirred Yogurt Manufacturing Fortified With Synbiotic EVOO Nanoemulsion and Nanocomposite Microcapsules

Fresh cow's milk was heated at 80°C for 15 min and cooled to 42°C (Fayed et al., 2019). The starter cultures of yogurt (*Lb.bulgaricus* and *S.thermophilus*) were added at the concentration of 2%. The inculcated milk was divided into the following treatments (Table 1). All microcapsules and probiotic-free cells were added with 2% for the treatments. The treatments were then transferred into plastic cups (100 ml) and incubated at 42°C for 4 h until coagulation. After that, the resulting yogurt was stirred, and the cups were kept at 7°C for 20 days.

**Table 1:** Treatments of stirred yogurt.

**Treatment**	**Starter**	**Probiotics-free**	**Nanoemulsion**	**Nanocomposite**
	cultures*	cells**	**microcapsules**	**microcapsules**
T1	**√**	**-**	**-**	**-**
T2	**√**	**√**	**-**	**-**
T3	**√**	**-**	**√**	**-**
T4	**√**	**-**	**-**	**√**

* *Starter cultures of yogurt (Lb.bulgaricus and S. thermophilus).*

** *Probiotics-free cells (Lb. acidophilus and B. bifidum)*.

#### Microbiological Activities of Stirred Yogurt Treatments

The diluting pouring plate technique was used for enumerating microbes in the samples. In the first dilution, tri-sodium citrate (3%, w/v) was used. The followed serial dilution was prepared by saline solution. The microbial activity of samples was determined for the count of *B. bifidum* by an MRS agar medium supplemented with 2 gm/l sodium propionate and 3 gm/l lithium chloride (Fayed et al., 2019). The plates were incubated at 37°C for 72 h under anaerobic conditions. The *Lactobacillus acidophilus* counts were evaluated by the MRS agar medium supplemented with 0.02% bile salts, and the plates were incubated at 37°C for 48 h under anaerobic conditions (Gilliland and Walker, 1990). The count of *S. thermophilus* was evaluated by the M17 agar medium, and the plates were incubated under aerobic conditions at 37°C for 48 h (IDF, 1997). Also, the counts of *Lb. bulgaricus* were determined using the MRS agar medium with pH 5.5 and the plated incubated under the anaerobic condition at 37°C for 48 h (IDF, 1997). Finally, the counts of mold and yeast were detected by potato dextrose agar acidified to pH 3.5 with a sterile lactic acid solution (10%). The plates were aerobically incubated at 25°C for 4 days (APHA, 1994).

#### Chemical Evaluation of Stirred Yogurt Treatments

Fresh stirred yogurt treatments were analyzed for dry matter, protein, fat, and ash contents (AOAC, 2012). The titratable acidity was assessed as illustrated by Ling (1963). The pH values were measured for yogurt from the different treatments using a digital pH meter (Hanna, Germany.

#### Sensory Evaluation of Stirred Yogurt Treatments

Stirred yogurt treatments were sensory evaluated by Ladokun and Oni (2014). The ratings were scored on a Hedonic scale, ranging from 1 to 5, for appearance, odor, mouth, feel, and overall acceptability. Keys: 5.0 = very good, 4.9–4.0 = good, 3.9–3.0 = fair, 2.9–2.0 = poor, 1.9–1.0 = bad. The evaluation was carried out by a regular 15-member scoring panel of the Dairy Department, National Research Center.

### Evaluation of the Antioxidant Activity by DPPH Assay

The DPPH free radical scavenging assay was used for the measurement of antioxidant activity of the developed microcapsules and prepared yogurt. ***In the*** ***case of microcapsules***, 3 g of dried milled microcapsules was dispersed in 100-ml methanol for 1 h and then filtered using Whatman No. 1 filter paper. The residue was re-extracted two times with additional 100-ml methanol for 15 min. Then, 5 μl of the combined filtrate was added to 2-ml methanol and 95 μl of freshly prepared 0.13-mM DPPH solution in methanol. The mixture was vigorously shaken and incubated for 30 min at room temperature in the dark. After incubation, the absorbance was measured at 517 nm against a blank (methanol) using a UV-Vis spectrophotometer (T80 UV/VIS Spectrometer, PG Instruments Ltd, UK) (A_sample_). The control was prepared by replacing the test sample with 5-μl methanol (A_control_). ***In the case of stirred yogurt***, 2.5-ml distilled water was added to 10 g of stirred yogurt (pH was set to 4 by adding 1-M HCl). The stirred yogurt was incubated at 45°C for 10 min and then centrifuged at 10.000 rpm for 10 min at 4°C. The supernatant was separated, and the pH was set to 7 by adding NaOH. Centrifugation was done again at 10.000 rpm for 10 min at 4°C, and about 250 μL of the sample was added to 2.75 ml of the methanolic solution of DPPH and stirred vigorously. Later, it was stored for 5 min at the ambient temperature, and the absorption was recorded at 517 nm (A_sample_). In the control sample (A_control_), distilled water was used instead of yogurt water (Tavakoli et al., 2018). The percentage of free radical-scavenging capacity was calculated by the following equation:


$$\begin{array}{l} \text{Radical}\;\text{scavenging}\;\text{capacity}\;\left(\%\right)=\;\frac{{\text{A}}_{\text{control}}-{\text{A}}_{\text{sample}\;}}{{\text{A}}_{\text{control}}}\text{X}\;100 \end{array}$$

All measurements were performed in triplicate and reported as the average value.

### Statistical Analysis

The data were analyzed using Statistical Analysis System Users Guide SAS (2004) (SAS Institute, Inc., USA). All data were carried out in triplicate, and mean values were calculated.

## Results and Discussion

### Physicochemical Analysis of EVOO

The physical and chemical analysis of EVOO was evaluated according to recommended methods. Peroxide value is an indicator of initial lipid oxidation. PV is 3.8 meq.O_2_/kg oil, which is lower than the legal limit of PV <20 meq.O_2_/kg oil, (EC, 1989/2003). Specific absorption coefficient K_232_ and K_270_ is indicative of the formation of conjugated dienes and trienes, respectively. The value of K_232_ and K_270_ is recorded at 1.79 and 0.16, respectively. The EU regulation established a value of K_232_ < 2.5 and K_270_ < 0.25 for extra virgin olive oil. The free acidity directly affected the flavor, color, stability, and quality of the olive oil. According to literature, free fatty acidity is a measure of the quality of the oil and reflects the care taken in production and storage processes of the oil, its stability, and its susceptibility to rancidity (Cobzaru et al., 2016). The acidity value is 0.15% that is below the maximum limit established for EVOO (≤ 0.8%).

The iodine value of investigated oil was found to be 90.75 g I_2_/100-g oil. This value is a measure of the content of unsaturated fatty acids present in the oil. A higher value indicates a higher degree of unsaturation. The saponification value is an indication of the molecular weights of triglycerides in oil. A higher saponification value indicates a low proportion of lower fatty acids because the saponification value is inversely proportional to the average molecular weight or chain length of the fatty acids. Therefore, the shorter the average chain length, the higher is the saponification number (Atinafu and Bedemo, 2011). The saponification value of extra virgin olive oil was determined to be 189-mg KOH/g oil. All the physicochemical parameters for EVOO meet the International Olive Council (IOC) certification criteria for EVOO (IOC, 2015).

### Droplet Size and Zeta Potential Analysis

**Figure 2:**
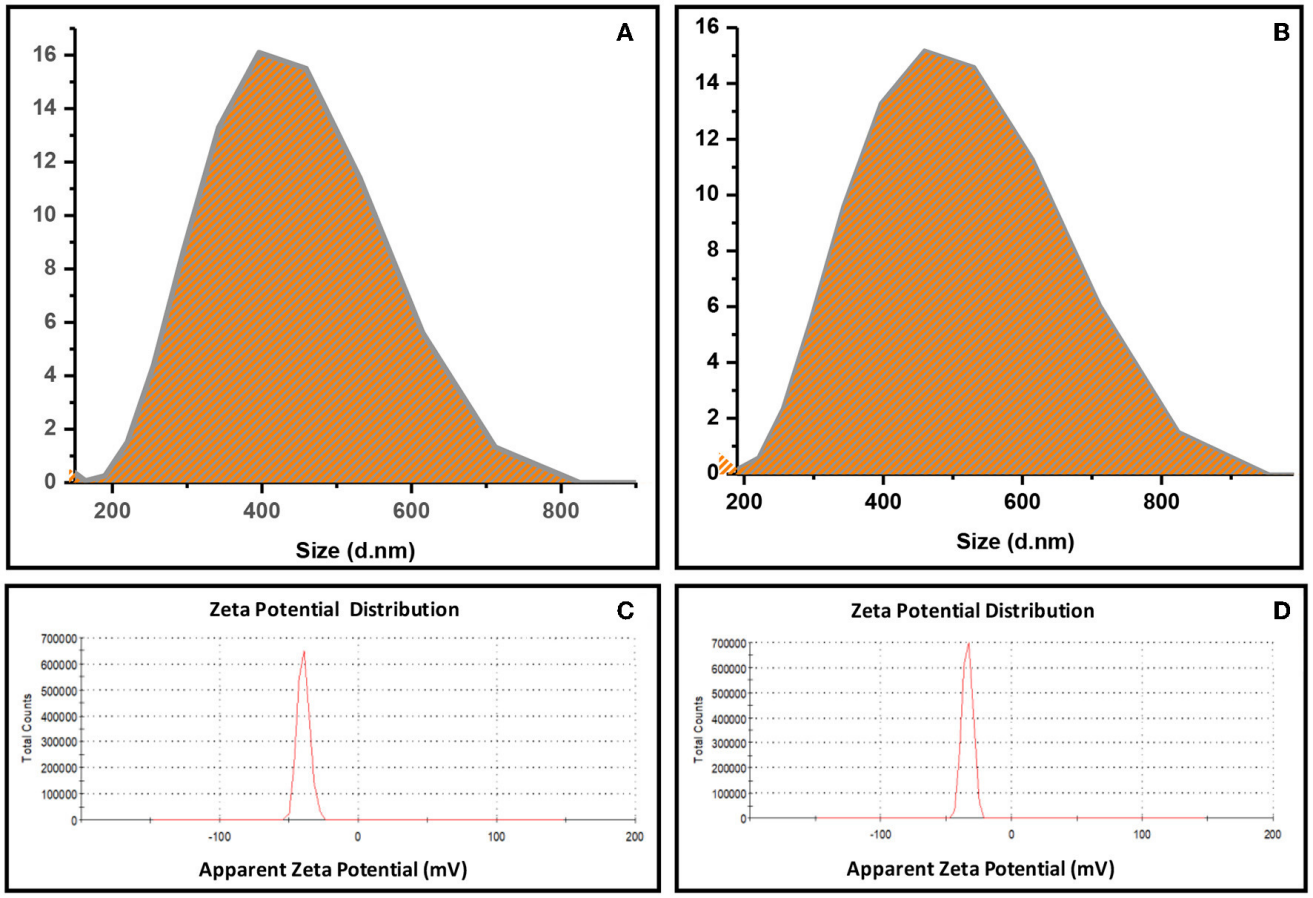
Droplet size distribution of **(A)** EVOO nanoemulsion, **(B)** EVOO nanocomposite and Zeta potential distribution of **(C)** EVOO nanoemulsion, **(D)** EVOO nanocomposite.

EVOO nanoemulsion and nanocomposite (Section First Stage: Preparation of EVOO Nanoemulsion and Nanocomposite) were measured by Malvern Zetasizer to determine the droplet size and zeta potential distribution (Figure 2). EVOO nanoemulsion showed a smaller-mean droplets size of around 416 nm than EVOO nanocomposite, which has a mean droplets size of about 475 nm. In addition, the zeta potential of EVOO nanoemulsion and nanocomposite was −39.6 and −33.6 mV, respectively. In general, the two formulations have a high negative value of zeta potential charge above −30 mV, which indicates that both nanoemulsion and nanocomposite are stable (Mcclements, 2007). The zeta potential measures the electrokinetic potential of a droplet and defines the stability of the formulation system. The system is considered stable when the value is more than +25 mV and lower than −25 mV (Che Sulaiman et al., 2016). McClements (2005) confirmed that nanoemulsion having a negative zeta potential and well dispersion of droplets has good stability.

### Oxidative Stability

The Rancimat method is officially recommended and is most commonly used for oxidative stability assessments of edible oils and fats (Ciemniewska-Zytkiewicz et al., 2014). The Rancimat test measures resistance to accelerated oxidation, which is a very important parameter for evaluating the quality of oils once it gives a good perception and estimation of the susceptibility to the oxidation process. The oxidative stability of EVOO extracted from prepared microcapsules in terms of measurement of induction periods (Rancimat, 20 L/ h at 110°C) was evaluated. The induction period (IP) of EVOO extracted from nanocomposite microcapsules (8 h) is lower than the IP of nanoemulsion microcapsules (11.30 h). During the extraction process of EVOO from nanocomposite microcapsules, polyphenols in EVOO (antioxidants) may be adsorbed onto nano-clay. Allaoui et al. (2020) used different solvents to recover the polyphenols after adsorption onto natural clay (ghassoul). Oxidative stability is not dependent on a single parameter but is rather affected by the fatty acid composition and a complex pool of antioxidants and prooxidants (Almoselhy, 2021).

### Microencapsulation Efficiency (ME)

The microencapsulation efficiency is the main factor in evaluating the effective encapsulation method and indicating the efficiency of the chosen encapsulating materials (Bora et al., 2019). The ME _probiotic_ was recorded as 88.84% for nanoemulsion microcapsules and 98.49 % for nanocomposite microcapsules. The nanocomposite microcapsules were more effective in the protection of probiotic strains inside microcapsules. This reason may be related to nano-clay material used in the preparation of nanocomposites microcapsules. Additionally, maltodextrin inside microcapsules acted as a prebiotic agent that maintained the viability of probiotic cells inside microcapsules either in the nanoemulsion or nanocomposite model (Bitaraf et al., 2013; Paim et al., 2016; Bhagwat et al., 2020; El-Sayed et al., 2021). Generally, the use of different microencapsulation techniques gave a positive impact on the viability of probiotic strains and maintained their viability for a long time as mentioned by other authors (El-Shafei et al., 2018; Chaudhary and Patel, 2019; Pourjafar et al., 2020).

Microencapsulation efficiency was evaluated by the degree of protection of the oil within the formed microcapsules. ME is the most essential factor in determining the success of oils microencapsulation, which is an indicator of a surface and coated oil content. As the wall material covered more oil droplets, the microencapsulation efficiency is higher. In the case of the nanocomposite microcapsule, ME_EVOO_ was 72%, while, in the nanoemulsion, the microcapsule was 65.61%. It can be seen that the ME value was affected by the addition of nano-clay. So, the nanocomposite microcapsule model had lower surface oil content compared to the nanoemulsion microcapsule model. According to the results, the combination of nano-clay with SA-WPC could increase the efficiency of protection of core oil. Singha and Hedenqvist (2020) reported that the barrier properties of coating composition were improved after the addition of nano-clay due to its platelet structure.

### Morphological Features

The morphology of the developed synbiotic EVOO nanoemulsion and nanocomposite was observed by TEM. From the TEM micrographs, the spherical shape of the oil droplets was distributed uniformly and homogenously without any aggregation throughout the formulated nanoemulsion or nanocomposite (Figures 31A,2A). Moreover, the droplet size analyzed from TEM corresponded with the size obtained from the Zetasizer analysis. It can be also noted that probiotic bacteria were appeared and coated with the used polymers (SA-WPC) in synbiotic EVOO nanoemulsion (Figure 31B). In the synbiotic EVOO nanocomposite, the coated probiotic bacteria were well dispersed between clay layers (Figure 32B).

**Figure 3:**
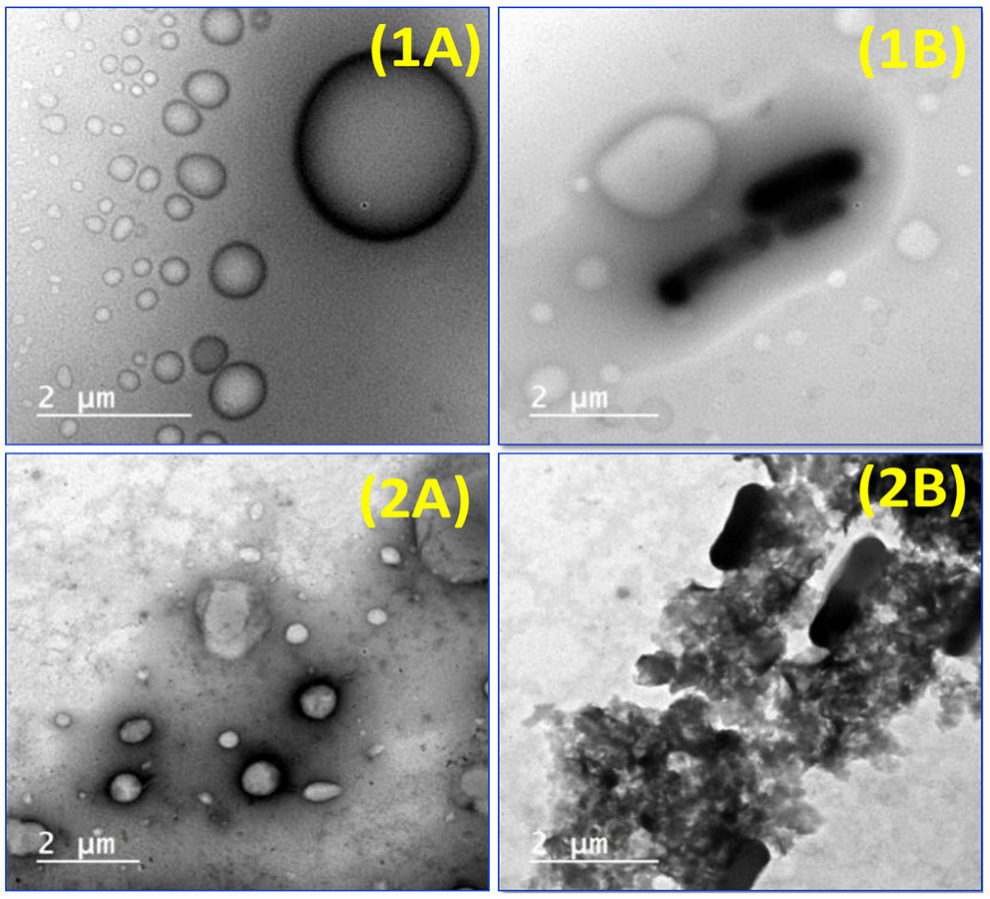
TEM micrographs of **(1A)** EVOO nanoemulsion **(1B)** Synbiotic EVOO nanoemulsion **(2A)** EVOO nanocomposite and **(2B)** synbiotic EVOO nanocomposite.

**Figure 4:**
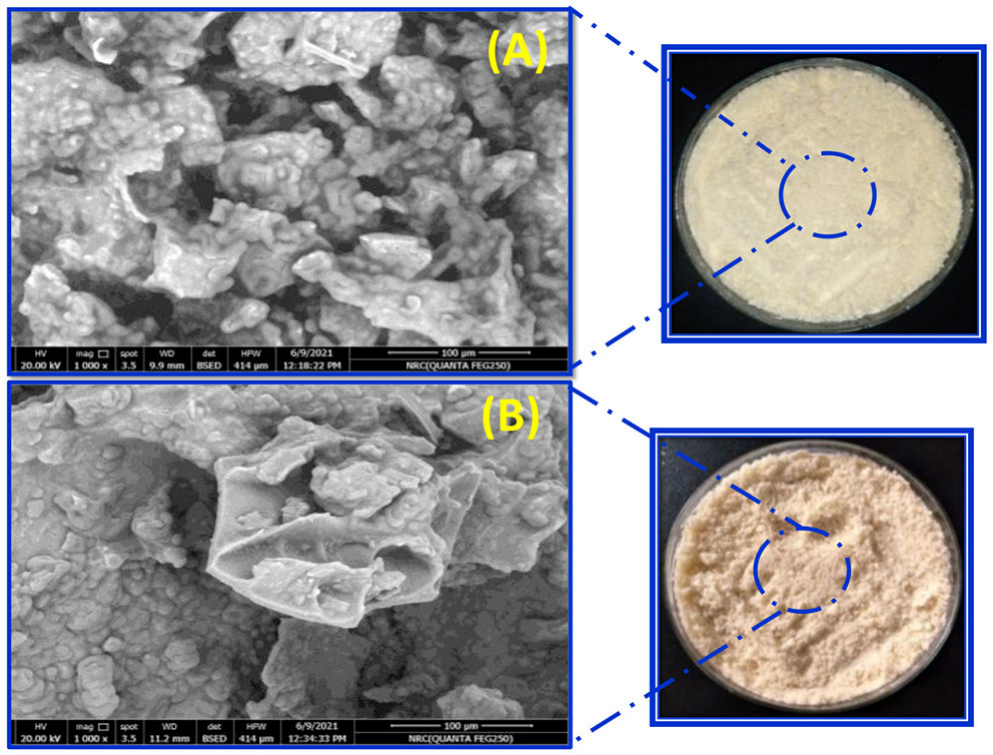
SEM micrographs of **(A)** synbiotic EVOO nanoemulsion microcapsules and **(B)** synbiotic EVOO nanocomposite microcapsules.

When examining the appearance of microcapsules models, it was seen that nanoemulsion microcapsules presented a bright white color. While the nanocomposite microcapsules were shown as beige, it may be due to the color of the nano-clay. The surface morphology of the nanoemulsion and nanocomposite microcapsules was investigated using SEM (Figure 4). As apparent from SEM photographs, freeze-dried microcapsules have crumpled: irregular shapes with a wide particle-size distribution and a porous structure. In the nanocomposite microcapsules, the overlapped structure of the clay was opened, and the distance between layers of the clay was observed (Figures 4, 3B). In the same context, Anwar and Kunz (2011) presented evidence that microcapsules obtained by freeze-drying had an irregular, very light, and porous structure compared to those produced by spray drying. Considering the proceeding results, the existing morphology may affect the release behavior of EVOO and probiotic bacteria.

### Release Evaluation and Survivability for Microencapsulated EVOO and Probiotic Strains

#### *In vitro* Survivability of Probiotic Strains Inside Microcapsules in Simulated Gastrointestinal Conditions

The viability of probiotic strains in gastrointestinal conditions is important with the intention to get the preferred benefits of probiotics. Firstly, free and microcapsules cells were exposed to gastric juice. An instant decrease was examined in free cells in contrast to the encapsulated cells with nanoemulsion and nanocomposite microcapsules (Table 2). The nanocomposite microcapsules showed a 0.63 log reduction, while the nanoemulsion microcapsules showed a 1.10 log reduction as compared to free non-encapsulated cells; the reduction was around (2.28 log) after 2 h in gastric juice. The microcapsules of the probiotic cells within either nanoemulsion or nanocomposite microcapsules significantly affected (*p* ≤ 0.05) the survivability of probiotic strains than free probiotic cells. Secondly, the remained free and microcapsules cells were exposed continuously to intestinal juice where the free cells and microcapsules were gently separated from gastric juice by cooling centrifugation and transferred to intestinal juice for additional 6 h. Different microcapsules models showed a protective effect on probiotic cells when exposed to intestinal conditions. From the result, a rapid log reduction was observed for free cells as compared to the encapsulated probiotics (Table 2).

**Table 2:** The survivability of microencapsulated probiotic strains particles in simulated gastrointestinal conditions.

**Encapsulation type**	**Initial count**	SGJ*(h)		SIJ*(h)			**Survival rate (%)**
		**1**	**2**	**2**	**4**	**6**	
Nanoemulsion microcapsules	9.10^Aa^	8.00^Ba^	7.50^Cb^	7.20^Ca^	7.00^Da^	6.30^Ea^	69.23^a^
Nanocomposite microcapsules	9.50^Aa^	8.87^Ba^	8.49^Ba^	7.58^Ca^	7.00^Da^	6.54^Ea^	68.84^b^
Probiotic free cells	9.18^Aa^	6.90^Bb^	5.85^Cc^	5.16^Cb^	4.60^Db^	3.37^Eb^	36.71^c^

* *SGJ, stimulated gastric juice; SIJ, stimulated intestinal juice. The viable count expresses as Log CFU/g beads. Rows with the same capital letter are not significant (p ≤ 0.05). Columns with the same small letter are not significant (p ≤ 0.05)*.

The microencapsulated cells in either nanoemulsion or nanocomposite models had a significant outcome on cell survivability. The survivability rate after exposure to gastrointestinal juices for 6 h was recorded at 69.23, 68.84, and 37.71% for nanoemulsion microcapsules, nanocomposite microcapsules, and free cells, respectively. The results confirmed that the microencapsulated probiotic strains with prebiotic maltodextrin using biopolymers (SA-WPC) are a useful tool for the longevity of sensitive cells and other compounds. The current findings were in accordance with Pimentel-Gonza'leza et al. (2009) encapsulated *Lb. rhamnosus* by the double-emulsion technique (w/o/w), and this process increased the resistance to the culture when exposed to conditions similar to those found in gastric juice and the presence of the bile.

#### EVOO and Probiotic Strains Release

##### The Release of Probiotic Strains

**Figure 5:**
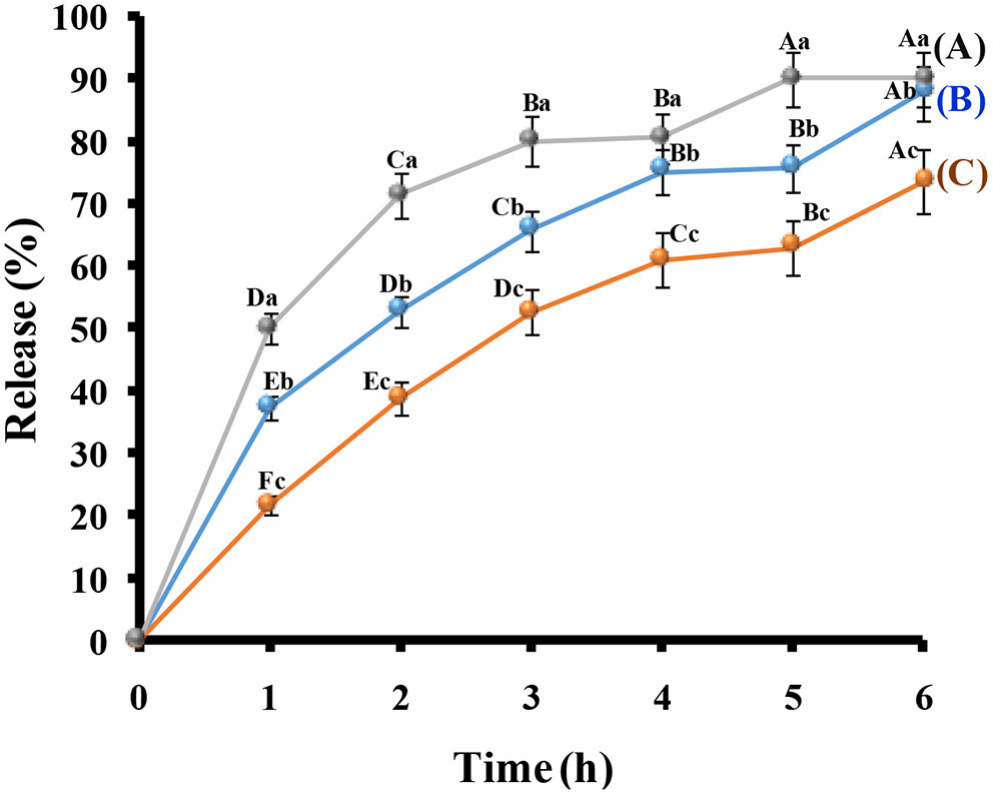
Probiotic strains released from microcapsules (A) probiotic-free cells, (B) nanoemulsion microcapsules, and (C) nanocomposite microcapsules. The capital letters between the release time and the same capital letters are not significant (*p* ≤ 0.05). The small letters between the free cells and microcapsules and the same small letters are not significant (*p* ≤ 0.05).

The time-dependent release of probiotic strains in microcapsules models and free cells in the simulated colonic solution is shown in Figure 5. In the first hour, more than 50% of the free cells were released from the dialysis bag, but about 30 and 20% of cells with nanoemulsion and nanocomposite microcapsules were released, respectively. Similarly, after 3 h, more than 50% of cells inside nanocomposite microcapsules were released. But, at the same time, more than 60% of the cells inside nanoemulsion microcapsules were released. Likewise, more than 80% of free cells were released from the dialysis bag at 3 h. The primary release for these preparations can be explained by the easy erosion of loose networks (Lotfipour et al., 2012). The release rate for cells was relatively remained stable with the same percentage for 4 h, and, after that, more released cells were observed. Also, in the case of microcomposite, the probiotic cells were aggregates and adhered to the surface of the clay, which may slow the release of the cells than nanoemulsion microcapsules (Li et al., 2014).

##### *In vitro* Release of EVOO

**Figure 6:**
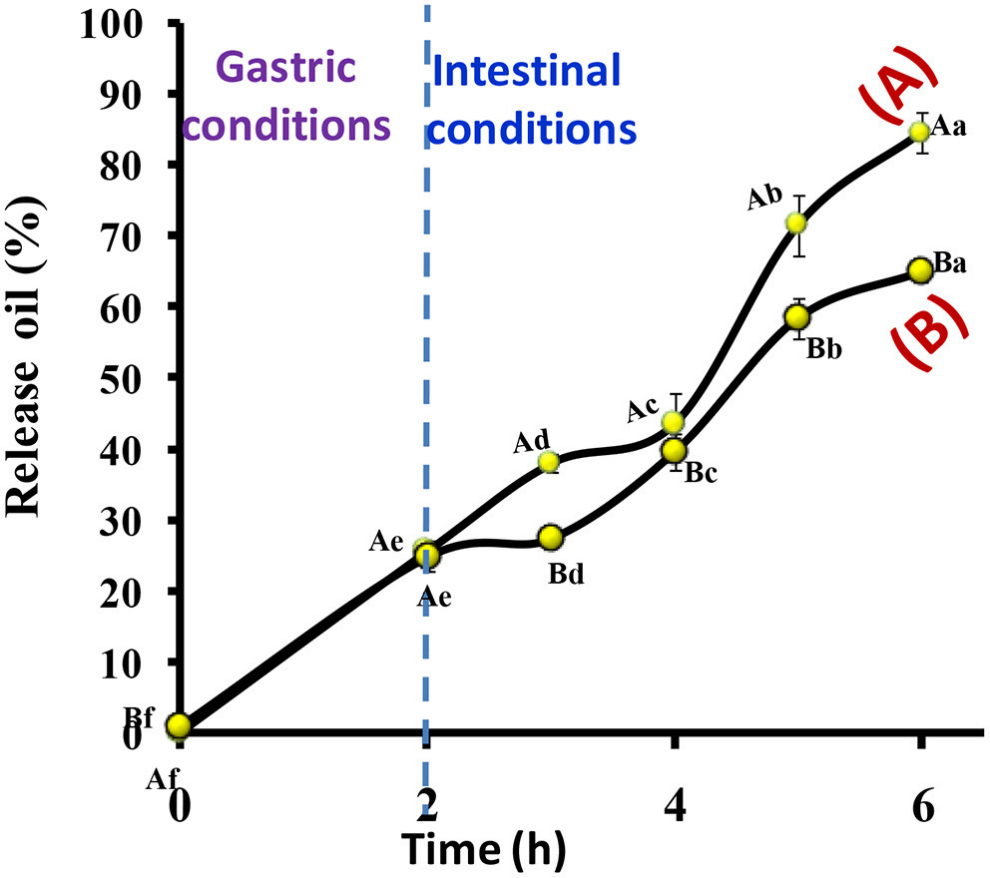
The release profile of EVOO from (A) nanoemulsion microcapsules and (B) nanocomposite microcapsules. The capital letters between microcapsules and the same capital letter s are not significant (*p* ≤ 0.05). The small letters between release time and the same small letters are not significant (*p* ≤ 0.05).

*In vitro* release profiles of EVOO from the prepared microcapsules were evaluated in gastrointestinal solutions at 37°C (Figure 6). The release properties of the prepared microcapsules were determined as a function of time. It was noted that nanocomposite microcapsules showed a slower release than nanoemulsion microcapsules. At gastric conditions, approximately 25% of EVOO were released from nanoemulsion and nanocomposite microcapsules in 2 h. The release at intestinal conditions was about 84 and 65% in 4 h from these microcapsules. Zhang et al. (2020) showed the release of imidacloprid and retarded by incorporating it into bentonite-alginate composites. The wall materials of microcapsules had a controlling effect on the release of EVOO as a core material. A similar trend was observed in the release behavior of the fish oil as a core of microcapsules (Pang et al., 2017). In addition, the release rate curve of the microcapsule gradually flattened with time, indicating that both EVOO and probiotic strains were successfully embedded and slowly released.

### Stirred Yogurt Manufacturing Fortified With Microcapsules Models

#### Microbiological Activities of Stirred Yogurt Treatments

The microbiological evaluation of stirred yogurt fortified with different microcapsules models and free cells as control is shown in Table 3. The count of *B. bifidum* in different treatments was significantly boosted during the storage period. But the count of *B. bifidum* in the T2 that contained free cells was increased in the first 10 days, and, after that, slight decline was observed at the end of storage and reached 6.14 log CFU/ml. Additionally, the count of *B. bifidum* in the microcapsules treatments was increased during the storage period, and the maximum count was observed at 15 days where the *B. bifidum* in the T3 and T4 was enhanced in the count with about 1.00 and 1.09 log cycles, respectively. From the results, the microcapsules models were able to maintain and enhance the viability of probiotic strain inside stirred yogurt than free cells (Paredes et al., 2016; Marefati et al., 2021).

**Table 3:** Microbiological evaluation of stirred yogurt treatments.

**Treatments**	**Zero**	**5**	**10**	**15**	**20**
***B. bifidum*** **counts (log CFU/mL)**					
T1	N.F	N.F	N.F	N.F	N.F
T2	6.96^Bb^	7.44^Ab^	7.84^Ab^	6.59^BCb^	6.14^Cc^
T3	7.33^Ba^	7.59^Ba^	8.10^Aa^	8.30^Ab^	8.27^Ab^
T4	7.48^Ba^	7.76^Ba^	8.33^ABa^	8.58^Aa^	8.40^Aa^
***Lb. acidophilus*** **counts (log CFU/mL)**					
T1	N.F	N.F	N.F	N.F	N.F
T2	7.00^Bb^	7.70^Ab^	7.91^Ac^	7.20^Cb^	6.85^Db^
T3	7.90^Ba^	8.58^Aa^	8.88^Ab^	8.60^Aa^	8.80^Aa^
T4	7.87^Ca^	8.78^Ba^	9.00^Aa^	8.82^ABa^	8.85^ABa^
***S. thermophilus*** **counts(log CFU/mL)**					
T1	7.29^Ca^	7.56^Cc^	8.28^Ab^	7.90^Bc^	7.55^Ba^
T2	7.39^Ba^	7.87^Bb^	8.40^Aa^	8.00^Aa^	7.22^Ca^
T3	7.33^Ba^	7.57^Bc^	8.51^Aa^	8.17^Ab^	7.25^Ca^
T4	7.36^Ba^	8.83^Aa^	8.50^Aa^	8.23^Aa^	7.19^Bb^
***Lb. bulgaricus*** **counts (log CFU/mL)**					
T1	6.85^Ba^	7.30^Aa^	7.55^Ab^	6.40^Cb^	6.28^Cc^
T2	6.91^Ca^	7.65^Aa^	7.90^Ab^	7.41^Ba^	6.85^Cb^
T3	6.96^Da^	7.58^Ba^	7.95^Aa^	7.60^Ba^	7.20^Ca^
T4	6.87^Ca^	7.55^Ba^	8.00^Aa^	6.67^Cb^	7.00B^Ca^
**Mold and Yeast counts (Log CFU/mL)**					
T1	N.D	1.90^Da^	2.20^Ca^	3.00^Ba^	4.98^Aa^
T2	N.D	N.D	N.D	2.10^Bb^	3.30^Ab^
T3	N.D	N.D	N.D	1.39^Ac^	2.53^Ac^
T4	N.D	N.D	N.D	2.90^Bb^	3.00^Ab^

*Data expressed as 3 replicates. T1, plain-stirred yogurt; T2, stirred yogurt with probiotic- free cells; T3, stirred yogurt with synbiotic EVOO nanoemulsion microparticles; T4, stirred yogurt synbiotic EVOO nanocomposite microcapsules; N.F., not found; N.D., not detected. Rows with the same capital letters are not significant (p ≤ 0.05). Columns with the same small letter are not significant (p ≤ 0.05)*.

Likewise, the count of *Lb. acidophilus* in stirred yogurt was enhanced during the storage period in the treatments (T2 and T3) than in the treatment with free cells (T2). The maximum viable counts for encapsulated *Lb. acidophilus* were observed on Day 15 of storage and slightly decreased with non-significantly recorded on Day 20 of storage. Generally, the *Lb. acidophilus* counts in T3 and T4 were enhanced by about 0.94 and 0.92 log cycles at the end of storage. In contrast, in the T2 that contained free cells, the viable counts slightly declined at the end of storage (6.85 logs CFU/ml). Our results are in the same line as Chávarri et al. (2010) and El-Sayed et al. (2015), who indicated that encapsulated probiotic strains with prebiotic materials were more stable and viable than free cells during storage in dairy products.

The starter cultures used in the manufacturing of yogurt were evaluated during storage as Table 3. The count of *S. thermophilus* in treatments was having the same activities and counts. Where the count of *S. themophilus* in the fresh stirred yogurt was recorded in 7 log cycles, the counts were improved for 15 days to reach 8 log cycles. But the decline in the *S. thermophilus* counts was detected on Day 20 (at the end of storage time), which is related to the acidity development during storage.

Moreover, the counts of the *Lb. bulgaricus* had the same activities, which not detected significant differences in their counts for all treatments during the storage period. The counts of *Lb. bulgaricus* in treatments were ranged between 6.0 and 7.0 log cycles during fresh and Day 20 of storage. But slight enhancement at 10 days of storage was observed, where the counts in all treatments were recorded between 7.5 and 8.0 log cycles.

The counts of mold and yeast were detected in stirred yogurt treatments during storage (Table 3). The data did not detect counts for mold and yeast in the fresh samples, which were related to the hygienic rules and pasteurization that follow during manufacturing. During storage, small mold and yeast counts were detected firstly in plain treatment (T1) on Day 5 of storage. Also, in the other treatments, the count of mold and yeast was detected with the storage time on Day 15 of storage, which may be related to the antimicrobial activities of probiotic strains that are integrated into these treatments (T2, T3, and T4). At the end of storage, the mold and yeast counts were reached 4.98, 3.30, 2.53, and 3.00 log CFU/ml for T1, T2, T3, and T4, respectively. So, our data recommended that the stirred yogurt shelf life did not exceed the 15 days of storage as mentioned by authors Yoon et al. (2013) that found the shelf life of yogurt beverages at 10, 15, and 25°C was as 19, 14, and 12 days, respectively, and 17, 16, and 12 days for stirred yogurt, respectively.

#### Chemical Evaluation Stirred Yogurt Treatments

The chemical composition of stirred yogurt treatments was found in Table 4. The total solid (T.S.) was ranged between 17.08 and 17.72%, and the high total solid content was indicated in T3 and T4, which contains microcapsules. Also, the moisture content was slightly different in all treatments, and little moisture content was found in treatments integrated with microcapsules (T3 and T4). Generally, moisture content was ranged between 82.28 and 83.13. The change in the total solid and moisture content in the T3 and T4 that are integrated with microcapsules may be related to found coating materials (SA-WPC) and the prebiotic agent (maltodextrin). Additionally, the results were detected light differences but non-significantly in total protein, which ranged between 3.85 and 4.05%.

**Table 4:** Chemical composition of stirred yogurt treatments.

**Treatments**	**T.S**	**Moisture**	**Protein**	**pH**	**T.A**
T1	16.08^B^	83.62^A^	3.85^B^	4.45^A^	0.99^B^
T2	17.48^A^	82.52^A^	3.95^B^	4.34^B^	1.01^A^
T3	17.53^A^	82.47^B^	4.03^A^	4.35^B^	1.02^A^
T4	17.72^A^	82.28^B^	4.05^A^	4.37^B^	1.04^A^

*Data expressed as 3 replicates. T1, plain-stirred yogurt; T2, stirred yogurt with probiotic-free cells; T3, stirred yogurt with synbiotic EVOO nanoemulsion microparticles; T4, stirred yogurt synbiotic EVOO nanocomposite microcapsules. Columns with the same capital letter are not significant (p ≤ 0.05)*.

The pH values in the treatments ranged between 4.34 and 4.45 with non-significantly differences, but a slight drop in the pH values was observed in the T1 that integrated with probiotic free cells. The development in the pH was fast in this treatment related to the metabolic activities of prebiotic free cells. Also, the titratable acidity (T.A.) was ranged between 0.99 and 1.04 and did not show significant differences between treatments. Our results are in the same line as Ribeiro et al. (2014); Fayed et al. (2019); Ismail et al. (2020) recorded that the pH values were lower in yogurt with free probiotics than in yogurt with encapsulated probiotics. Chouchouli et al. (2013) designed the study with the direct fortification of plain yogurts, producing stirred fortified yogurts.

#### Sensory Evaluation of Stirred Yogurt Treatments

**Figure 7:**
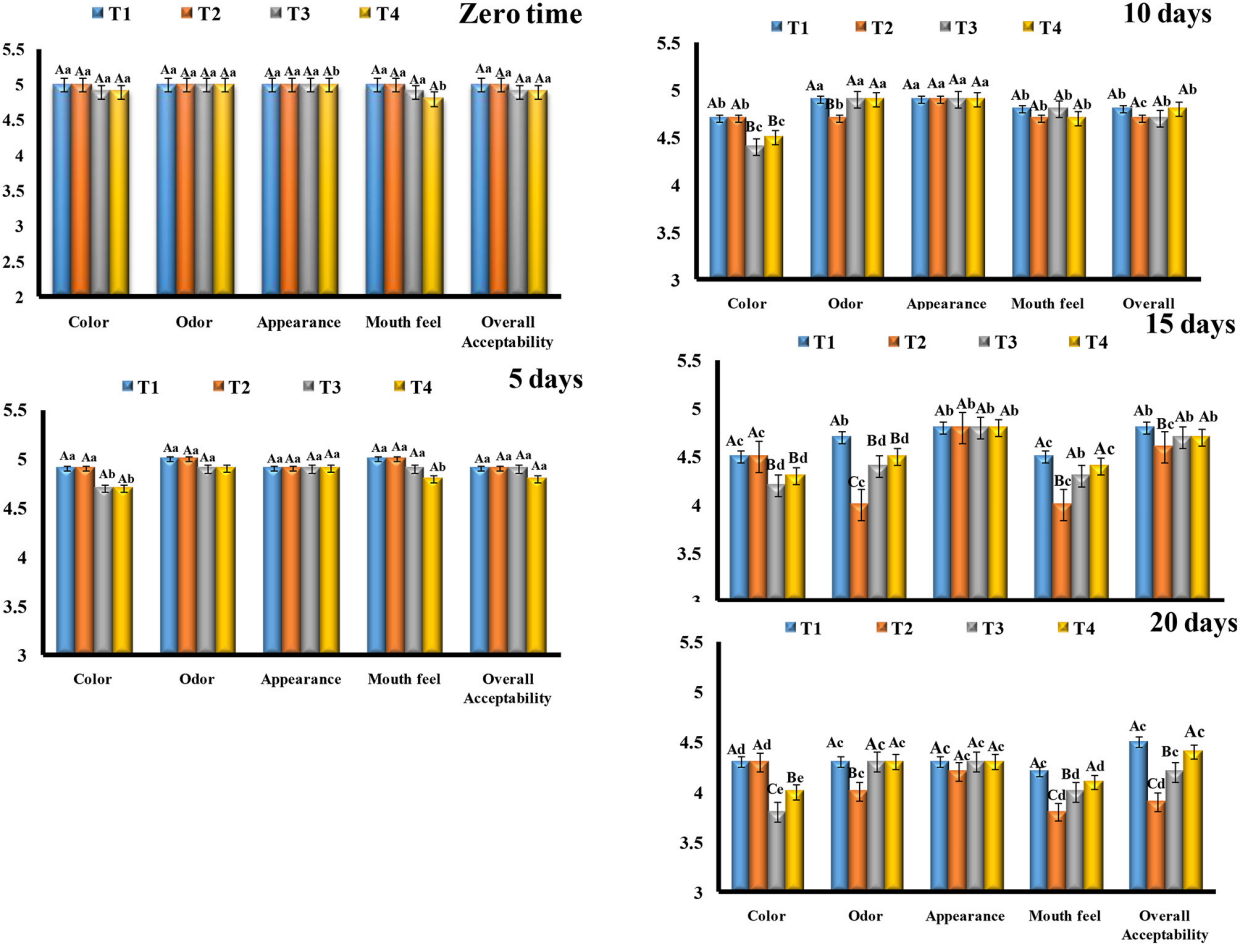
Sensory evaluation of stirred yogurt treatments. The capital letters between treatments and the same capital letters are not significant (*p* ≤ 0.05). The small letters between storage time and the same small letters are not significant (*p* ≤ 0.05). Data expressed as (mean ± SD) of 3 replicates. T1, Plain-stirred yogurt; T2, stirred yogurt with probiotic-free cells; T3, stirred yogurt with synbiotic EVOO nanoemulsion microparticles; T4, stirred yogurt synbiotic EVOO nanocomposite microcapsules.

Sensory evaluations of stirred yogurt treatments during storage are shown in Figure 7. Fresh treatments even with microcapsules models or free cells displayed that plain-stirred yogurt and stirred yogurt with free cells (T1 and T2) were higher white than stirred yogurt with microcapsules models (T3 and T4) due to the yellow color of microcapsules powder that added. This yellow color could be related to the EVOO and other coating materials (SA-WPC). For odor and appearance, all treatments were very good and did not detect significant differences between them. On the other hand, the highest score for the mouth feel was observed for plain-stirred yogurt and stirred yogurt with free cells (T1 and T2) followed by stirred yogurt with nanoemulsion microcapsules and nanocomposite microcapsules (T3 and T4), respectively, which could be related to the feeling with small particles during evaluation by members (Kailasapathy, 2006; Ismail et al., 2020). Overall acceptability in fresh time was recorded as 5 for T1 and T2 but slightly low as 4.9 for T3 and T4.

During the storage period, the color and appearance were decreased in all treatments; this could be related to the moisture lost over time, as it made the treatments more yellow, especially for treatments with microcapsules models (T3 and T4). Moreover, the mouth feel of treatments was decreased by the time of storage, and more decrease was observed for stirred yogurt with free cells (T2), followed by stirred yogurt with nanoemulsion microcapsules (T3) at the end of storage (20 days). These results could be related to the development of the acidity faster in the treatment with free cells (T2) than others that are related to the metabolic activity of free cells than encapsulated cells.

The encapsulated materials were slow down the release of the acidity that produced from the microbial strains activities during storage period (Ribeiro et al., 2014; Afzaal et al., 2019). Generally, the overall acceptability for all treatments was decreased with the storage time, but the plain-stirred yogurt (T1) was the most treatment accepted all the time, followed by stirred yogurt with nanocomposite microcapsules (T4), stirred yogurt with nanoemulsion microcapsules (T3), and stirred yogurt with free cells (T2), respectively, at the end of the storage period. During storage, the score for sensory evaluation of stirred yogurt with free cells (T2) was dropped, and then microcapsule treatments (T3 and T4) could be related to a more sour feeling and an odor that developed from metabolic activities of strains during storage. In contrast, a more drop in the score for color was observed for treatments with encapsulated particles (T3 and T4). So, our data recommended that the suitable shelf life for the stirred yogurt with microcapsules that maintained the viability of probiotic strains not exceeds 15 days to be acceptable to the consumer as plain-stirred yogurt.

### Antioxidant Activity of Prepared Microcapsules and Stirred Yogurt Treatments

**Figure 8:**
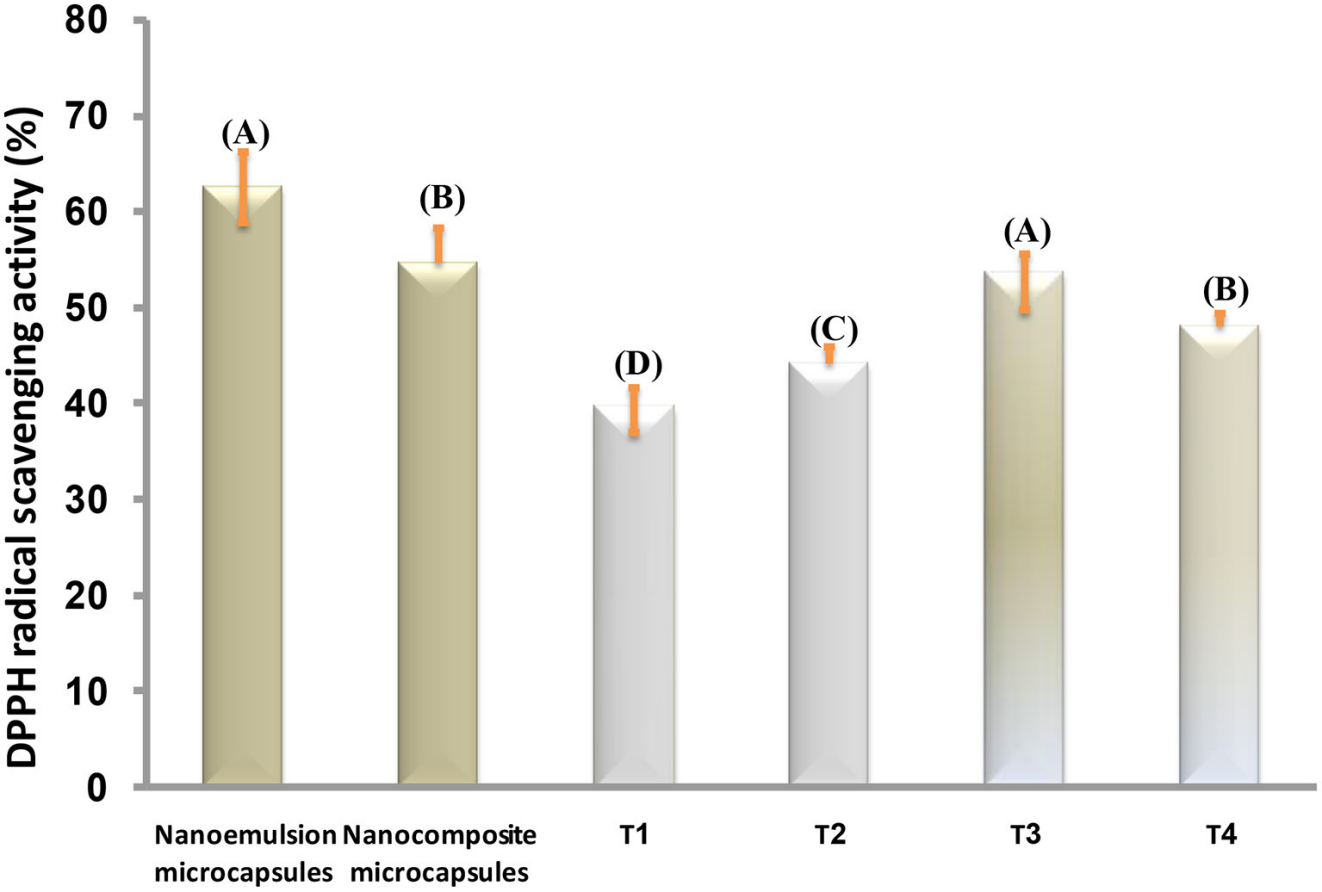
DPPH radical scavenging activity (%) of microcapsules and stirred yogurt treatments. The same capital letters are not significant (*p* ≤ 0.05). T1, plain-stirred yogurt; T2, stirred yogurt with probiotic-free cells; T3, stirred yogurt with synbiotic EVOO nanoemulsion microparticles; T4, stirred yogurt synbiotic EVOO nanocomposite microcapsules.

The antioxidant activity (AA) of prepared microcapsules and stirred yogurt was determined by the DPPH radical scavenging assay. The DPPH scavenging activity assay is widely used to evaluate the ability of compounds to scavenge free radicals or donate hydrogen and to determine the antioxidant activity in foods (Bidchol et al., 2011). EVOO is a source of phenolic compounds with a powerful antioxidant activity that includes tyrosol, hydroxytyrosol, and secoiridoid derivatives (Francisco et al., 2019). According to Figure 8, the antioxidant activity values of the nanoemulsion microcapsules were higher than nanocomposite microcapsules. In stirred yogurt, T1 showed AA possibly due to the presence of compounds in milk, such as low molecular weight antioxidants, free amino acids, lactose, peptides, proteins, or reducing compounds (Oliveira and Pintado, 2015). Microcapsules-fortified yogurts exhibited higher AA than plain-stirred yogurt. The addition of nanoemulsion microcapsules to stirred yogurts increased antioxidant activity more than plain yogurt (T1). It was noted that T4 has a little higher AA (48%) than T2 (44%). Ribeiro et al. (2014) showed that powder-fortified yogurts exhibited higher antioxidant activity than the control. The oxidative stability was higher for nanoemulsions than for nanocomposite microcapsules, which was consistent with the AA results.

## Conclusion

Stirred yogurt was fortified with two models of microcapsules based on synbiotic EVOO nanoemulsion and nanocomposite. These microcapsules were prepared in three stages: nano emulsification, blending, and freeze-drying technique. EVOO nanoemulsion showed a smaller mean droplet size than EVOO nanocomposite, and both nanoemulsion and nanocomposite had good stability. The nanoemulsion microcapsules model displayed better oxidative stability than the nanocomposite model. However, the nanocomposite microcapsules were more effective in the protection of EVOO and probiotics than the nanoemulsion model. The morphology of the nanoemulsion and nanocomposite that showed the spherical shape of the oil droplets was distributed uniformly and homogenously without any aggregation. It can be also noted that probiotic bacteria appeared and coated with the used polymers (SA-WPC). From SEM photographs, the two models of microcapsules have crumpled: irregular shapes with a wide particle-size distribution and a porous structure. The nanocomposite microcapsules showed a slower release of EVOO and probiotics than nanoemulsion microcapsules. Additionally, probiotic bacteria inside developed microcapsules were enumerated with good active counts after being exposed to gastrointestinal juices. Finally, the addition of nanoemulsion microcapsules to stirred yogurts increased antioxidant activity more than plain yogurt. Moreover, the viability of probiotic strains in microcapsules form was more active in the stirred yogurt during storage. The most accepted treatment in the sensory evaluation was recorded as the plain-stirred yogurt, followed by stirred yogurt with nanocomposite microcapsules and stirred yogurt with nanoemulsion microcapsules. Overall, stirred yogurt was an appropriate delivery carrier for microencapsulated active probiotic bacteria and EVOO.

## Data Availability Statement

The original contributions presented in the study are included in the article/supplementary material, further inquiries can be directed to the corresponding author/s.

## Author Contributions

HE-S and AH conceived the research idea and helped to collect the data. KY, HE-S, and AH analyzed the data and wrote the paper. All authors contributed to the article and approved the submitted version.

## Conflict of Interest

The authors declare that the research was conducted in the absence of any commercial or financial relationships that could be construed as a potential conflict of interest.

## Publisher's Note

All claims expressed in this article are solely those of the authors and do not necessarily represent those of their affiliated organizations, or those of the publisher, the editors and the reviewers. Any product that may be evaluated in this article, or claim that may be made by its manufacturer, is not guaranteed or endorsed by the publisher.
